# Parasitic strongyle nemabiome communities in wild ruminants in Sweden

**DOI:** 10.1186/s13071-022-05449-7

**Published:** 2022-09-27

**Authors:** Peter Halvarsson, Paulius Baltrušis, Petter Kjellander, Johan Höglund

**Affiliations:** 1 Department of Biomedical Sciences and Veterinary Public Health, Swedish University of Agricultural Sciences, 7036, 750 05 Uppsala, Sweden; 2 Department of Ecology, Swedish University of Agricultural Sciences, Grimsö Wildlife Research Station, 739 93 Riddarhyttan, Sweden

**Keywords:** Nemabiome, Metabarcoding, Helminth, Gastrointestinal parasites, Ungulates, Invasive species, Biodiversity

## Abstract

**Background:**

Wildlife hosts may serve as reservoirs for strongyles, which can be transmitted to domestic livestock. Therefore, studies evaluating nemabiome compositions in wildlife ruminants are of great use in assessing the possibility of transmission of important nematode pathogens to domestic sheep in Sweden.

**Methods:**

First, fecal samples were collected from roe deer (*n* = 125), fallow deer (*n* = 106), red deer (*n* = 18) and mouflon (*n* = 13) in south central Sweden during the hunting season in 2019. Second, after fecal examination samples were cultured and the larvae were harvested, followed by DNA extractions. Third, all samples were barcoded and processed for sequence analysis on the PacBio platform. Finally, bioinformatic sequence analysis was conducted with DADA2, while species diversity and richness, as well as interactions between the different hosts, were calculated and analyzed in R.

**Results:**

Nematode ITS2 sequences were found in 225 of 262 (86%) samples. In total, 31 taxa were identified, among which 26 (86%) to the species level. These were found in different combinations, among which 24 (77%) occurred in roe deer, 19 (61%) in fallow deer, 20 (65%) in red deer and 10 (32%) in mouflon. Five of the species found are known to be associated with livestock (*Chabertia ovina, Haemonchus contortus*, *Oesophagostomum venulosum, Teladorsagia circumcincta* and *Trichostrongylus axei*). However, in the present study the relative abundance and prevalence of most of these species were low. The most striking exception was *T. axei*, which was relatively abundant in all wildlife hosts. Mostly a wide range of wildlife specific nematodes such as *Ostertagia leptospicularis* and *Spiculopteragia* spp. were identified including the invasive nematode *Spiculopteragia houdemeri*, which was found for the first time in red deer, fallow deer, and mouflon in Sweden. The difference in the number of shared species between mouflon and all cervids (*n* = 6) was less than among all three cervids (*n* = 8).

**Conclusion:**

In this study, we investigated the community structure of parasitic intestinal nematodes in four wildlife hosts, and we found that the majority of the parasite species identified were wildlife specific. We also found a new, potentially invasive species not reported before. After comparing the nemabiome of the wildlife hosts in this study with a previous study in sheep from the same geographical region, we conclude that the horizontal transmission potential appears to be relatively low. Still, cross-infections of nematodes between game and sheep cannot be completely ignored.

**Graphical Abstract:**

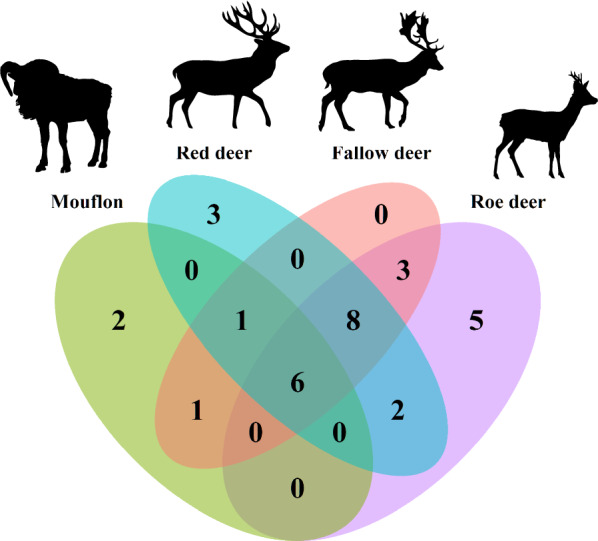

## Background

The interest in helminths of wildlife has increased in recent years because of possible economic implications for domestic livestock. First, it has been established that several wild ungulates can act as reservoirs of generalist parasites, which in turn may be cross-transmitted to livestock [1–3]. Second, it has been suggested that climate change may have consequences affecting the transmission biology of parasites between wildlife and domestic animals. For example, in the case of roe deer (*Capreolus capreolus*) expanding populations and habitat change have altered the host range [4]. With a general trend towards warmer and wetter grazing seasons, this may eventually lead to increased exposure to pathogens in livestock when different host species interact [5]. Third, altered exposure can also be an outcome of increased trade of infected game animals between distant regions. Some examples are the recent introductions of *Ashworthius sidemi* and *Spiculopteragia houdemeri* into Europe. Both species are regarded as invasive parasites originating from Asia and have been spreading in Central Europe since the second half of the twentieth century [6–9].

When it comes to nematode infections in small ruminants, attention is for obvious reasons primarily paid to naturally occurring pathogens such as *Haemonchus contortus*, *Teladorsagia circumcincta* and *Trichostrongylus axei* [3, 10]. All three are important abomasal strongyle nematodes in domestic sheep, among which in particular *H. contortus* and *T. axei* have been recorded in different combinations especially from roe deer (*Capreolus capreolus*) across Europe [11–20], but also in fallow deer (*Dama dama*) in Poland [18], as well as in red deer (*Cervus elaphus*) in Italy and Poland [18, 21]. Both *H. contortus* and *T. circumcincta* [22] are nowadays commonly found in sheep flocks in the south-central part of Sweden while the occurrence of *T. axei* is more sporadic, as shown in a nemabiome study conducted on samples collected from 61 commercial farms [23]. Although *H. contortus*, which is globally considered the single-most important pathogenic parasite in sheep [22], was uncommon in Swedish roe deer in the past, it has been suggested for decades that this parasite could be transmitted from wildlife hosts to sheep [24]. However, it is unknown how widespread this species is in roe deer today and whether transmission via other wild hosts takes place. However, there is experimental evidence [25], as well as a genetic study of specimens collected from different hosts in the Alpine area, suggesting that transmission between wild and domestic ruminants do occur [26]. Similarly, it has been shown that *T. axei* is a cosmopolitan generalist showing high rates of gene flow between sympatric host species [27]. In addition to these nematodes, *Chabertia ovina* and *Oesophagostomum venulosum,* can also be transmitted between the wild ruminants and sheep [3]. However, according to general knowledge these two species are unusual and not considered major pathogens for sheep in Sweden.

Because it is well documented that wild ruminants share some nematodes with domestic livestock, they can theoretically also transmit worms carrying anthelmintic resistance determining alleles as well as susceptible genotypes [28]. Along with the emerging levels of anthelmintic resistance in European ruminant livestock nematodes, mainly associated with *H. contortus* and *T. circumcincta* [29], the role of wildlife as vectors of resistant strains has been proposed. While Chintoan-Uta et al. [17] concluded that roe deer have the protential to acquire resistant *H. contortus* from livestock, Brown et al. [30] suggested that wildlife hosts could contribute equally to delay the spread by acting as an untreated source of refugia. This was further substantiated in a Hungarian study in which *H. contortus* was shared by sheep and roe deer but the homozygous susceptible genotype was more common in the latter [19]. Thus, even if the transmission of resistant *H. contortus* genotypes between domestic and wild animals has been verified experimentally on a shared pasture, it is not guaranteed to occur out on farms [25].

First, the likelihood of cross-transmission of parasites between different host species is affected by the density of infected animals on shared pastures where they share the same resources [3, 31]. Although some parasites are known to infect closely related hosts, it is at the same time well established that there may be differences in susceptibility and parasite fecundity in different host species. For example, according to an experimental study, European mouflon were shedding > 20,000 *H. contortus* eggs per gram of feces 11 weeks after infection [25]. In addition, the developmental and survival capacity of the parasites' free-living stages plays an essential role as these are key in the transmission process. For instance, in a Canadian investigation of the ecology of the free-living stages of strongyles in cattle revealed that a large number of larvae remained in the fecal pats at the end of grazing season but short-term rainfall had an important effect on the migration of larvae on pasture [32]. As a result, pasture-borne parasites show seasonal patterns of infection, which are highly sensitive to both climate change and land use. Like other organisms, nematodes are adapted to the conditions in the local environment [33]. Thus, the sensitivity of the free-living stages of different species have evolved differently as a response to temperatures and humidity levels in the environment. For example, the infective larvae of some species, such as *Ostertagia* spp. and *Trichostrongylus* spp., are in general cold-adapted and can overwinter on the pasture if not ingested during the first year [34], whereas others, such as *H. contortus*, appear sensitive to temperatures < − 3 ℃, even though this particular species is spread across the Holarctic region [35]. In short, the risk for cross-transmission of nematodes between the wildlife and domestic hosts is also likely to be influenced by the overwintering strategy employed by different parasite species.

Traditionally, nematode communities in wildlife have been identified by morphological criteria in adult male worms recovered at necropsy. With the advances in molecular technologies, it is nowadays possible to utilize fecal samples to determine the genera and/or species present. Recently, a method for simultaneously identifying all possible strongyles, which are the most abundant and diverse parasites in livestock ruminants, has been developed based on next-generation sequencing of the internal transcribed spacer 2 rDNA amplicon [36]. This technology has also been utilized to survey samples from roe deer in France [15] as well as from other wild ungulates in the USA [28]. The most important advantages of this approach are increased sensitivity and specificity as well as the unbiased quantification of whole parasitic nematode communities and alleviation of problems associated with cryptic species [36].

Currently, there is only limited knowledge about helminths occurring in Swedish wildlife as few nationwide and systematic studies have been conducted with the focus on parasitic nematodes. To the best of our knowledge, there are only two published studies: the first is based on adult worms removed from the ingesta of roe deer (*n* = 306) and moose (*Alces alces*) (*n* = 19) conducted in the late 1960s [24], while the second is based on samples from dead or debilitated moose (*n* = 50) [37]. The present investigation focuses on deer species in areas where most sheep farms are located in Sweden—roe deer, fallow deer and to a certain extent also red deer and mouflon (*Ovis aries musimon*). The three cervids are fairly common in Sweden, but mouflon is rare [38]. Among these, roe deer has the highest abundance in Sweden and has occurred for > 100 years throughout the whole sampling area. It is the only cervid on the island of Gotland. Fallow and red deer have increased on the mainland during the last 30 years in the southern parts of the country (www.viltdata.se, hosted by Swedish Hunters Association, 2022). Mouflon is considered an exotic species in Sweden, and the latest population estimation conducted in 2005 estimated the population to roughly 1000 animals [38, 39]. In the present study we investigated the strongyle nemabiome communities in the said wildlife hosts to provide baseline data to better understand and assess the risk for an exchange of parasites between wild and domestic ruminants.

## Materials and methods

### Samples

**Fig. 1:**
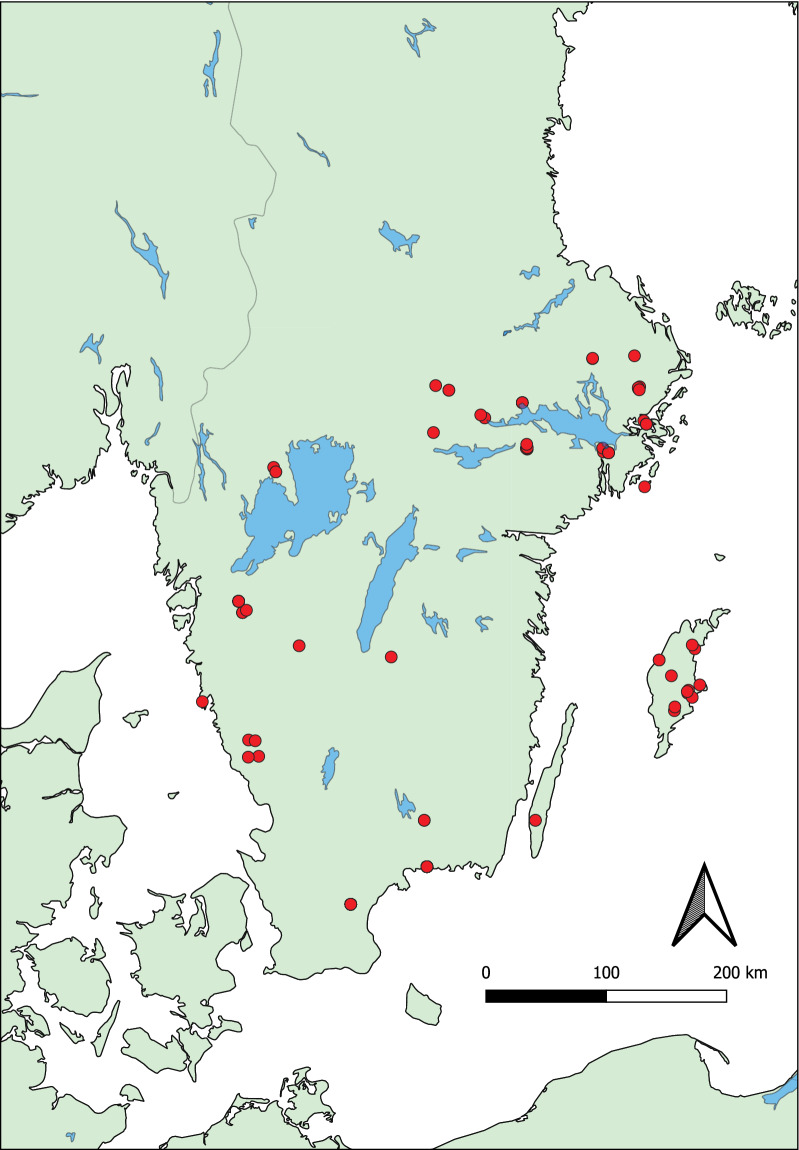
Map over sampling locations in southern Sweden. On the island of Gotland (to the left in the figure), only roe deer are present

Ahead of the main hunting season from August to November 2019, hunters were informed about the study via hunting press and asked to participate. Those that agreed to participate were instructed how to collect and store (+ 4 ℃) fecal samples from the rectum of red deer, fallow deer, roe deer and mouflon in airtight zip-lock bags prior to sending them to the laboratory. After the samples were received at our laboratory by post or by direct submission, they were stored short term at + 4 ℃ prior to further processing. Together with an additional 29 roe deer samples from Vidilab AB, we received in total 18 red deer fecal samples, 106 fallow deer samples, 125 roe deer samples and 13 mouflon samples. The sites where the animals were (hunted and) sampled were distributed throughout southern Sweden (Fig. 1). Fecal egg counts were analyzed using a modified McMaster protocol [40]. Coprocultures were set using the remaining feces, and L3 larvae were collected using the inverted Petri dish method as described earlier [41]. In addition to these samples, we also received L3 coprocultures from a commercial diagnostic laboratory. Total DNA was extracted from the larvae using the Nucleospin DNA tissue kit (Macherey-Nagel) according to the manufacturer's protocol.

### Molecular methods

Each sample was amplified using the universal nematode internal transcribed spacer region 2 (ITS2) ribosomal DNA primers (NC1-NC2), which were combined with unique 8-bp barcodes prior to pooling them for sequencing. In short, 50-µl PCR reactions were performed in duplicate and cleaned up using AMPure XL magnetic beads. The cleaned-up PCR products were pooled in equal amounts prior to sequencing on Pacific Biosciences sequencing platform with SMRT cell V3 RSII at SciLifeLab, Uppsala, Sweden. For further details, see Halvarsson and Höglund [23].

### Bioinformatic analysis

Each of the sequencing pools were demultiplexed using lima v2.4 (https://github.com/PacificBiosciences/barcoding) (lima reads.fq.gz barcodes.fasta demux.fastq -hifi-prefix SYMMETRICS). DADA2 [42] package in R was used here to infer amplicon sequence variants (ASV) from the sequenced dataset. First, reads containing unresolved nucleotides were removed (maxN = 0). Second, primers on both ends were removed from the amplicon sequences (dada2::removePrimers). Third, sequences with a higher-than-expected error number (maxEE = 2) and sequences < 200 bp were removed while the sequencing error rates (learnErrors, errorEstimationFunction = PacBioErrfun) were estimated and used to correct the dataset. Fourth, sample data were dereplicated (derepFastq). Fifth, samples were inferred with the dereplicated dataset as input data (dada). Finally, chimeras were removed [removeBimeraDenovo(method = ‘consensus’)] and taxonomic assignment of ASVs was performed (assignTaxonomy) using the taxonomic nematode ITS2 database (v1.2.0), downloaded from https://www.nemabiome.ca/its2-database.html [36, 43]. To account for contaminations, singleton reads of each ASV, as well as ASVs with a read count of < 0.5% of the total, per sample, were filtered out [44]. Furthermore, samples with < 200 reads were removed from further analyses [23]. The Basic Local Alignment Search Tool (BLAST) available at GeneBank (https://www.ncbi.nlm.nih.gov/genbank/) was used to verify correct species assignment and to fill in missing taxonomic data for unresolved ASVs based on identity. ASV assignment to organisms other than parasitic nematodes were removed. Species assignment to an ASV was only considered if the identity percentage was ≥ 98.5% of the reference sequence. All ASV reads identified to the same taxonomic identity (i.e. species, according to the aligned sequence similarity to the reference database) were merged. This species clustering reduces hundreds/thousands of ASVs into a handful of species and is an important step in the analysis of nemabiomes. The more ASVs that are found belonging to the same species, the more genetically diverse that species is. For example, we grouped different ASVs into *Trichostrongylus* sp. A and *Trichostrongylus* sp. B based on sequence similarity and best matching sequence in our BLAST searches. Finally, the final species dataset was used for statistical analyses.

### Statistical analysis

All statistical analyses were conducted in R v4.2.0 (released on 2022-04-22) [45]. Species richness was calculated by summing up all species, and after standardizing the read counts based on the relative frequencies, inverse Simpson and Shannon-Wiener alpha diversity indices were calculated using the R package vegan v2.5.7. Package VennDiagram v1.7.1 was used to create the Venn diagram, whereas the UpSet plot was created with package ComplexUpset v1.3.3, where a cutoff of hosts was implemented at *n* = 3 and combined into Fig. 5. Plots were visualized using ggplot v3.3.5.

## Results

### Species diversity and richness

**Fig. 2:**
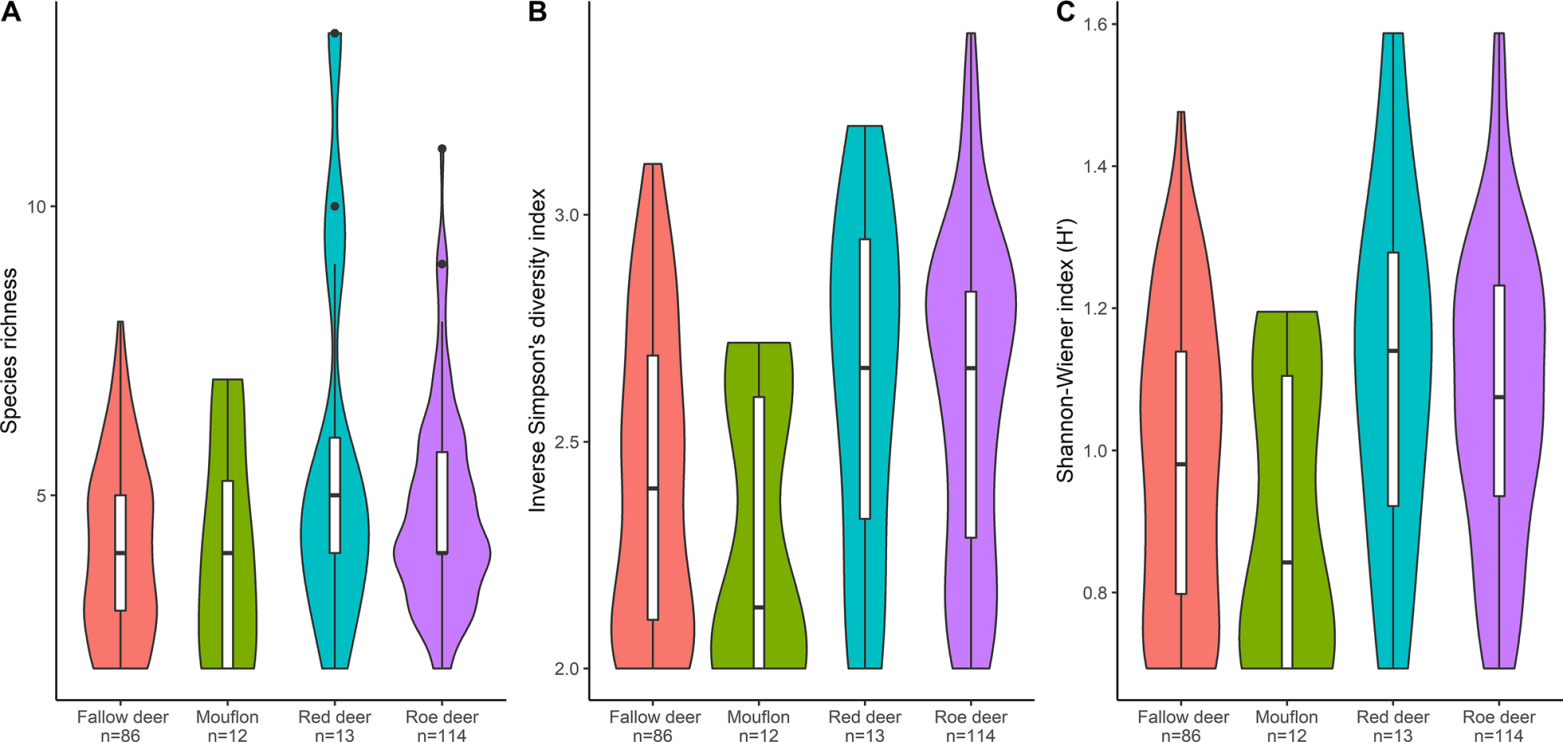
Parasite diversity plots for the four host species. Species richness for the four host species (**A**), where the highest number of parasite species was found in an individual red deer. Inverse Simpson’s diversity index (**B**) and Shannon–Wiener index H’ (**C**) are two different measurements of alpha diversity for the host species. Boxplots inside the violin plots display median values

**Fig. 3:**
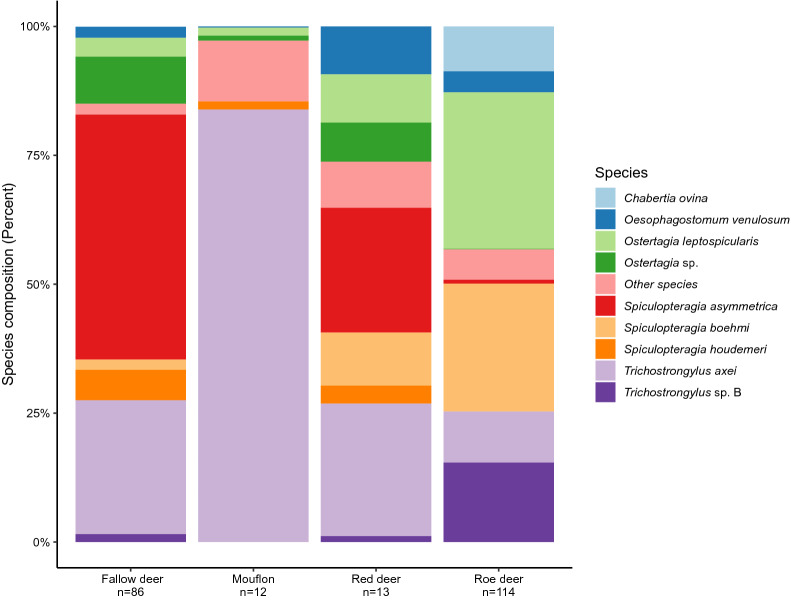
Species composition summary bar plot. Mean frequencies for the nemabiome composition for the four host species where *Trichostrongylus axei* is a common parasite in all hosts

After filtering and ASV clustering with DADA2, 13/18 red deer, 86/106 fallow deer, 114/125 roe deer and 12/13 mouflon samples were retained. In total, 884,113 reads were obtained from the samples (on average 3929, ranging between 202 and 7931 reads per sample). ASV clustering yielded 916 ASVs representing 31 nematode species among which 26 (84%) were identified to the species level. The greatest diversity was found in roe deer (*n* = 24), followed by fallow deer (*n* = 19), red deer (*n* = 20) and mouflon (*n* = 10), and the number of species differed significantly between the hosts (GLMSpecies richness, *F* = 4.44, df = 3, *P* = 0.0047). In addition, the mean species diversity in samples from various hosts, as measured by the two alpha diversity indices, were significantly different between the groups. Mouflon had the lowest diversity values, whereas red deer the highest (GLM_InverseSimpsons_, *F* = 6.88, df = 3, *P* =  < 0.0001; GLM_Shannon-Wiener_, *F* = 5.93, df = 3, *P* =  < 0.0001) (Fig. 2). Among the species identified nine dominated: *Chabertia ovina*, *Oesophagostomum venulosum*, *Ostertagia leptospicularis*, *Ostertagia* sp., *Spiculopteragia asymmetrica*, *Spiculopteragia boehmi*, *Spiculopteragia houdemeri*, *Trichostrongylus axei* and *Trichostrongylus* sp. B. Together these nine species represented 837464 (94.7%) reads and formed 627 (68%) ASVs (Table 1 and Fig. 3).

**Table 1:** Parasite species found in the four hosts and sequencing read counts for the parasites

Species	Roe deer (*n* = 114)			Fallow deer (*n* = 86)			Red deer (*n* = 13)			Mouflon (*n* = 12)				
	Read count	Individuals	Prevalence (%)	Read count	Individuals	Prevalence (%)	Read count	Individuals	Prevalence (%)	Read count	Individuals	Prevalence (%)	ASV count	GenBank ID
**Chabertia ovina**	47878	32	26	44	1	1							56	KF913470.1
*Cooperia oncophora*										33	1	8	6	AB534601.1
*Cooperia *sp.				33	1	1				66	1	8	1	AB534601.1
*Cylicocyclus ashworthi*	140	3	2										6	MW198060.1
*Cyathostomum catinatum*	990	5	4				12	1	6				16	MT193653.1
*Coronocyclus coronatus*	957	3	2	264	1	1							9	KY747448.2
*Cylicocyclus leptostomus*	133	1	1										4	KP693432.1
*Cylicocyclus nassatus*	3420	8	6	476	1	1	95	3	17				29	MT193649.1
*Cylicostephanus longibursatus*	1080	5	4	845	1	1							23	MW282937.1, MH483934.1
*Cylicostephanus calicatus*	233	2	2	137	1	1	41	1	6				14	MW367018.1, MW367002.1
*Cylicostephanus goldi*							66	2	11				4	KM085357.1
*Cylicostephanus minutus*	3900	2	2				7	1	6				15	MW282942.1, MH487659.1, MW282946.1
*Dictyocaulus *sp.	40	2	2	269	2	2	270	2	11				1	KF007339.1
*Elaphostrongylus rangiferi*							485	1	6				3	AF504027.1
*Haemonchus contortus*	1728	6	5	2692	2	2	83	3	17				45	LS997564.1
*Mazamastrongylus dagestanica*	7676	11	9										17	JQ925868.1
*Muellerius capillaris*										22	1	8	3	AY679527.1
*Oesophagostomum dentatum*	1357	3	2	200	3	3	93	2	11				17	KU891915.1
***Oesophagostomum venulosum***	16035	15	12	7896	5	5	4570	3	17	90	1	8	27	HQ283349.1
***Ostertagia leptospicularis***	132112	102	82	9469	23	22	4442	6	33	665	5	38	173	KC998722.1
*Ostertagia ostertagi*	30	1	1										2	AB245023.2, AB245008.2
***Ostertagia *sp.**	642	8	6	27630	52	49	2839	4	22	470	3	23	10	KC998722.1
***Spiculopteragia asymmetrica***	3576	15	12	165245	71	67	13357	9	50	278	1	8	44	AF480617.0, AF480617.1, MT322614.1
***Spiculopteragia boehmi***	113217	77	62	8459	9	8	2651	4	22				80	AJ577460.1
***Spiculopteragia houdemeri***				18011	27	25	2021	3	17	816	3	23	46	AB682702.1
*Strongylus vulgaris*							40	1	6				3	MF489225.1
*Teladorsagia circumcincta*	2238	1	1	866	5	5	2928	2	11	5271	8	62	36	MZ148615.1
***Trichostrongylus axei***	53263	48	38	82777	45	42	13476	9	50	43514	12	92	133	KC998725.1
*Trichostrongylus colubriformis*	3644	2	2										6	JF680985.1
*Trichostrongylus *sp* A*	388	2	2	3243	4	4	158	1	6				29	HQ844229.1
***Trichostrongylus *sp* B***	61356	62	50	611	4	4	54	2	11				58	KC998731.1
Total	456033			329167			47688			51225			916	

Read count = number of sequence reads obtained for the host species. For example, sequences were obtained for 114 roe deer (of 125 sampled). Individuals = number of infected hosts. Prevalence = percentage of host infected of a specific parasite divided by number of samples obtained. ASV count = number of ASV matching to a specific species. Genbank ID is the best match to the ASVs. The sum of all sequence reads for the parasite species in bold correspond to > 99% of the sequence reads

**Fig. 4:**
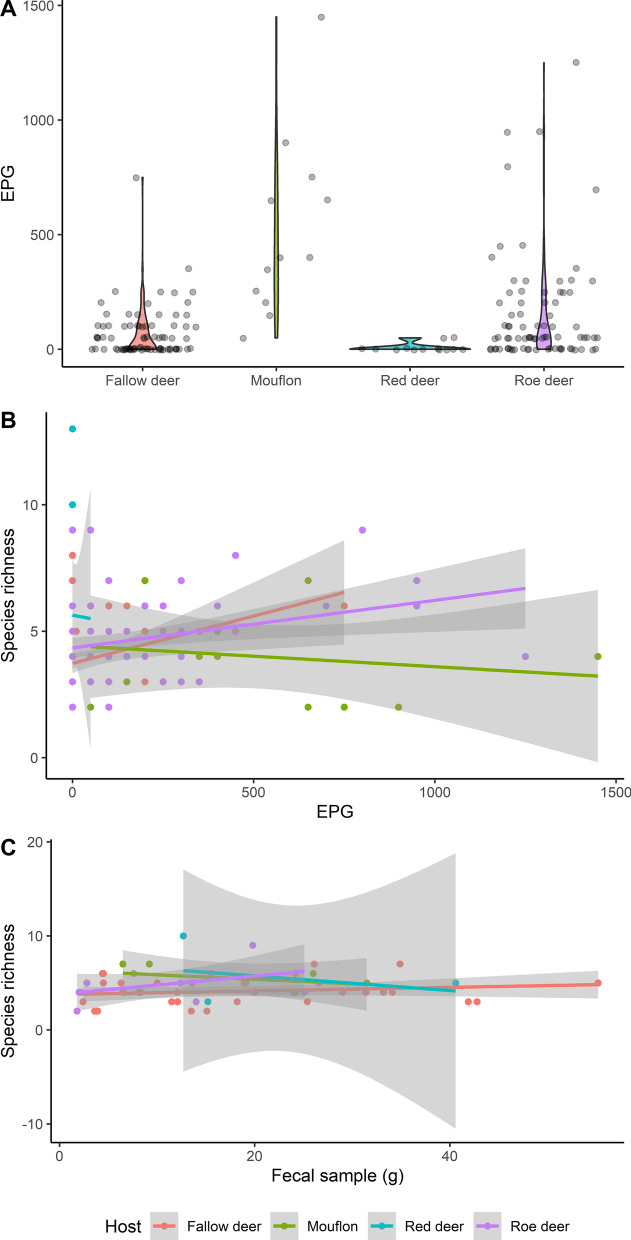
Effects of EPG and fecal sample on species richness. Most of the samples had low EPG, but mouflon had on average the highest EPG count (**A**). Species richness increased with higher EPG (**B**), but the fecal sample for coprocultures did not affect species richness (**C**)

In contrast, mouflon had the highest number of nematode eggs per gram (EPG) feces (mean EPG: 517, SD = 392), followed by roe deer (mean EPG: 152, SD = 237), fallow deer (mean EPG: 68, SD = 108) and red deer, which had the least (mean EPG: 7, SD = 19) (Fig. 4A). Parasite species richness was not affected by EPG (GLM: *t* = 28.74, df = 187, *P* = 0.086), nor was the amount of fecal sample processed for coproculture (GLM: *t *= 10.72, df = 54, *P* = 0.54) (Fig. 4B, C).

### Prevalence

The number of hosts infected with a particular species varied across host species. The three most prevalent species in roe deer were: *O. leptospicularis* (82%), *S. boehmi* (62%) and *Trichostrongylus* sp. B (50%); in fallow deer: *Ostertagia* sp. (49%), *S. asymmetrica* (67%) and *T. axei* (42%); in red deer: *O. leptospicularis* (33%), *S. asymmetrica* (67%) and *T. axei* (42%); in mouflon: *O. leptospicularis* (38%), *Teladorsagia circumcincta* (62%) and *T. axei* (92%). For details about the occurrence and relative abundance of all parasite species in each host species, see Table 1.

### Species based on ASV clusters

**Fig. 5:**
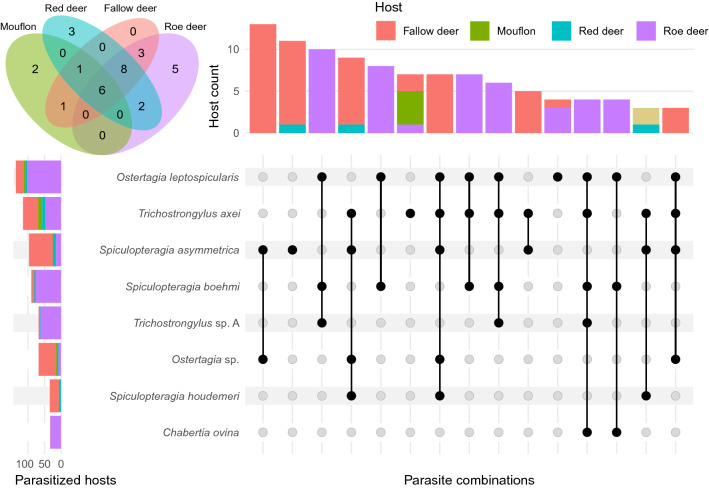
Host specificity and community composition. The Venn diagram in the upper left corner shows number of shared and unique parasite species in the four host species. Six species were shared by all hosts (*Oesophagostomum. venulosum*, *O. leptospicularis*, *Ostertagia* sp., *Spiculopteragia asymmetrica*, *Teladorsagia circumcincta* and *Trichostrongylus axei*). The upset diagram displays parasite combinations that can be found in at least three hosts, where the most common combination (*Spiculopteragia asymmetrica* and *Ostertagia *sp.) was found in 13 fallow deer hosts. The most commonly identified parasite, *Ostertagia leptospicularis*, was found in 136 hosts

After joining different ASVs belonging to the same species (i.e. performing species clustering) based on similarity from NCBI BLAST, we found 31 unique taxa. Six species (19%) were shared by all host species: *Oesophagostomum venulosum* (at low relative abundance in cervids, 2–9% and insignificant in mouflon, < 1%), *O. leptospicularis* (low to moderate in all hosts, 1.5–30%), *Ostertagia* sp. (insignificant in roe deer and mouflon, < 1%, and low levels in fallow and red deer, 8 and 9%), *S. asymmetrica* (insignificant in roe deer and mouflon, < 1% and moderate in fallow and red deer, 24 and 48%), *Teladorsagia circumcincta* (insignificant in all hosts, < 1%) and *T. axei* (high levels in mouflon, 84% but also low to moderate in the other hosts, 10–26%) (Fig. 5). *Trichostrongylus axei* was one of the most genetically diverse species based on number of ASVs. It also seems to be a generalist, as the 12 most common ASVs were found across all host species.

In cervids, 13 (42%) parasite species were shared in at least two of the host species, which were not found in mouflon. These were: *Chabertia ovina* (at low prevalence in fallow deer and moderate in roe deer, 1% and 26%), *Coronocyclus coronatus* (low prevalence in roe deer and fallow deer, 1% and 2%), *Cyathostomum catinatum* (low prevalence in roe and red deer, 4% and 6%), *Cylicocyclus nassatus* (low prevalence in roe and fallow deer, 6% and 1%, and moderate in red deer, 17%), *Cylicostephanus calicatus* (low prevalence in all, 1–6%), *Cylicostephanus longibursatus* (low prevalence in fallow deer and roe deer, 1% and 4%), *Cylicostephanus minutus* (at low prevalence in roe and red deer, 2% and 6%), *Dictyocaulus* sp. (low to moderate prevalence in all, 2–11%), *Haemonchus contortus* (low to moderate prevalence in all, 2–17%), *Oesophagostomum dentatum* (low prevalence in all, 2–11%), *S. boehmi* (at a high prevalence in roe deer, 62%, but at low to moderate in fallow and red deer, 8% and 22%), *Trichostrongylus* sp. A (low prevalence in all, 2–6%) and *Trichostrongylus* sp. B (at a high prevalence in roe deer, 50% but at low to moderate in fallow and red deer, 4–11%) (Table 1).

In addition, we identified ten species (32%) that were only found in one host species, among which two in mouflon (*Cooperia oncophora* and *Muellerius capillaris*), three in red deer (*Cylicostephanus goldi, Elaphostrongylus rangiferi, Strongylus vulgaris*) and five in roe deer (*Cylicocyclus ashworthi, Cylicocyclus leptostomus, Mazamastrongylus dagestanica, Ostertagia ostertagi, Trichostrongylus colubriformis*), while fallow deer had no unique species. (Fig. 5). However, most of these were uncommon and were only found in a few host individuals, except for *M. dagestanica*, which was found in as many as 11 of 125 (9%) and at an average relative abundance of 14% (varying between 0.1 and 98%) in roe deer.

## Discussion

Because wild ruminants can act as reservoirs for certain nematodes, they may play a key role in shaping the spatial distribution of nematode communities in domestic grazing livestock. In addition to the fact that knowledge of the biological diversity of parasites in wild hosts is of general biological interest, this justifies the study of the nemabiome composition of wild ungulates from a veterinary perspective. In the current study, which focuses on the role of certain wildlife acting as reservoirs for strongyle nematodes in sheep, we identified 31 species of which 24 (77%) in roe deer, 19 (61%) in fallow deer, 20 (65%) in red deer and 10 (32%) in mouflon, using nemabiome sequencing, performed on cultured fecal samples containing nematode larvae. Among the species identified, as few as 15 (48%) composed > 99% of the retrieved reads. The three most common species were: *O. leptospicularis, S. boehmi* and *Trichostrongylus* sp. B in roe deer; *S. asymmetrica, Ostertagia* sp. and *T. axei* in fallow deer; *O. leptospicularis*, *S. asymmetrica* and *T. axei* in in red deer and *O. leptospicularis*, *T. circumcincta* and *T. axei* in mouflon. When merged these accounted for 85% of the total number of reads. Of particular interest is that, in addition to *T. axei*, we also identified four species, which have recently been reported in domestic sheep, in the same geographical region [23, 44]. Among these, only *T. axei* was found at low to high levels in the wildlife hosts. In contrast, the relative abundance estimates for species known to occur in sheep (*C. ovina, H. contortus, O. venulosum* and *T. circumcincta*) were insignificant to low and/or absent in some wildlife hosts. Combined, these results suggest that investigated ungulates may play a role in the spread of parasitic nematodes in pastures where domestic livestock graze. However, since the nemabiome profiles in domesticated sheep and the studied wildlife hosts look so different, this seems unlikely to occur. Still, the risk of cross-transmission of for example *H. contortus* cannot be ignored.

As pointed out by Poulin and Mouillot [46], host specificity of helminth parasites increases with decreasing taxonomic distinctness between their host species. Of 31 species identified in our study, 21 (68%) occurred in more than one type of host, while 10 (32%) occurred in only one host species. However, only six species (*O. venulosum*, *O. leptospicularis*, *Ostertagia* sp., *S. asymmetrica*, *T. circumcincta* and *T. axei*) were found at variable relative abundances in all four wildlife hosts. This is in line with Wyrobisz-Papiewska et al. [47], who, based on a combined morphological-molecular approach, concluded that for example *O. leptospicularis* is a generalist in cervid and bovid hosts. Similarly, it has been shown that *T. axei* is a generalist [27]. On the other hand, six other species (*C. calicatus*, *H. contortus*, *O. dentatum*, *S. asymmetrica* and *Trichostrongylus* sp. B) were also identified, which were only shared by all cervids but not mouflon. Thus, according to our data, the number of species that were shared between the cervids were higher compared with those in mouflon. This is in agreement with [3], who stated that specialist helminths tend occur in a pair of closely related ruminant species. Although *O. leptospicularis* and *T. axei* were among the most frequently represented species in all hosts species included in the study, the cervids were more frequently infected with well-known nematodes that are wildlife specific, such as those within genus *Spiculopteragia* and two unidentified *Trichostrongylus* spp. In contrast, the few mouflons were mainly infected with nematodes that they share with sheep such as *Oesophagostomum* spp., *T. circumcincta* and *T axei*, although others such as *S. asymmetrica, S. houdemeri* and *Ostertagia* sp. were also shared with the deer.

Surprisingly, mouflon, unlike the cervids in the present study, was not infected with *H. contortus*, which is a parasite that mainly survives the winters inside its host as arrested larvae in Sweden [48]. As it is well known that mouflon is more susceptible to *H. contortus* than cervids [25], it is likely that we did not identify this species in our dataset because of limited sample size for this host (*n* = 12). Nevertheless, the observation is in line with Balicka-Ramisz et al. [49], who also did not find *H. contortus* in mouflon in an annual study conducted across Poland. On the other hand, it was prevalent in mouflon from both the alpine region in Italy [14] and in Spain [50]. Contrarily, we found *H. contortus* in roe, fallow and red deer. This finding is consistent with some studies [16, 18, 20, 21, 51] but not others, where between 20% to more than half of the examined animals were infected with *H. contortus* [11, 13, 17, 19]. Although the relative abundance of *H. contortus* was insignificant in all hosts, we found the prevalence in red deer was higher as opposed to roe and fallow deer. In addition, in agreement with the present study, it appears that *H. contortus* is in general rarer than *T. axei* in cervids [16, 20, 21, 52]. Interestingly, in the present study we found that *T. axei* was one of the most prevalent parasites with 38% of roe deer, 42% of fallow deer, 50% of red deer and 92% of mouflon being infected. This is in agreement with Bolukbas et al. (2012) and Chintoan-Uta et al. [17], who reported the prevalence estimates for *T. axei* in roe deer between 67 and 80%. Our prevalence figures for *T. axei* presented here (38%) are also higher than those reported previously for roe deer in Sweden (11%) [24], as well as in some other European countries [16, 18, 20]. Also, our present figures for red and fallow deer (50% and 42%, respectively) are higher than those in other studies (1–20%) [14, 17, 18, 21, 50, 53]. In any case, if the wild hosts in our investigation do act as reservoirs, it seems contradictory that *T. axei*, unlike *H. contortus*, is unusual in domestic sheep from the same region. In our study, we found that *T. axei* was the dominating species in mouflon, whereas it was far less prevalent in the deer hosts. The 12 haplotypes (ASVs) with highest read numbers were found in all host species supporting the view that *T. axei* is a true generalist. However, as suggested by Walker and Morgan [1], the actual transmission of nematodes between wildlife and livestock is not guaranteed simply by the fact that the same parasite species is present in multiple hosts. Population studies similar to those of Archie and Ezenwa [27], which examined the genetic variation also in other genetic regions than ITS2 of different isolates of the same parasite from several host species, are consequently required before more definitive conclusions about the actual role of wildlife hosts can be drawn.

Proper identification of the parasites in the different host species is of course fundamental to the understanding of the possibilities of cross-transmission between them. Even if some members, such as those in the superfamily *Trichostrongylidae*, at a first glance appear to be rather specific to a species or family of hosts, others are observed in a wide variety of host species. As suggested by Suarez and Cabaret [57], both host specificity and environment play significant roles in shaping the species composition even if the impact of each factor is not easily assessed. As it is sometimes difficult to distinguish closely related species solely on the basis of morphological characteristics, confirmation by molecular methods is usually required [54]. This is because there is strong evidence for the presence of morphs among several members in the family *Trichostrongylidae*. There is, for example, genetic evidence that *T. circumcincta*, *Teladorsagia trifurcata* and *Teladorsagia davtiani*, which have been described in a wide range of wildlife and domestic hosts, are a single species [55]. Genetic data also imply that *S. asymmetica* and *Spiculopteragia quadrispiculata* constitute morphologically distinct variants of a single species [56]. Similarly, it has been suggested that *O. colchidae* and *O. leptospicularis* represent a single species pair [57]. However, there is also strong evidence to suggest that *O. leptospicularis* is a cryptic species, as it has been demonstrated experimentally that the wildlife strain is distinctive from the bovid strain [47]. In addition, hybridization between closely related species sometimes occurs, for example as between *Haemonchus* spp. during communal grazing conditions in the tropics [58]. Combined, these phenomena (polymorphic and cryptic species, hybridization) seem common among trichostrongylid nematodes. This is illustrated in our study as the species identified were represented by multiple ASVs (see Table 1). This in turn complicates the comparisons of our present findings with those in previous prevalence studies on the species composition of nematodes in European cervids. This is because most European studies are based on traditional methods, except for one by Beaumelle et al. [15], in which the species identification in samples from roe deer was instead based on a similar nemabiome analysis approach.

When we compared our ITS2 nemabiome data set with those from roe deer in France [59], the outcomes mainly supported each other, but they also differed in some respects. For example, *C. ovina*, *O. leptospicularis*, *O. venulosum* and *T. axei* were among the most prevalent ones in both roe deer studies. In addition, only some animals were infected with *H. contortus* or *T. circumcincta* in both studies. One difference, however, is that while we identified both *S. boehmi* (prevalence = 62%; 25% of the reads) and *S. asymmetrica* (prevalence = 12%; 8% of the reads), these species are not identified in the French study. However, *S. boehmi* is a well-known parasite of roe deer in The Netherlands [16] and in Poland [18], which is in line with our finding. We also found that *S. boehmi* occurred in both fallow and red deer. While *S. asymmetrica* is usually the species in this genus associated with these two cervids, as in our study, it has also been described from roe deer [18, 53, 60, 61]. In fact, according to our analysis *S. asymmetrica* was represented by 50% of the reads in fallow deer and 28% of the reads in red deer. In addition, 4% to 5% of the reads in these hosts matched with *S. houdemeri*. Although this parasite is mainly known from a wide range of native cervids in the Far East, it has been described in great detail by both morphological and molecular tools from specimens recovered from sika deer (*Cervus nippon*) in Japan [62]. Recently, *S. houdemeri* has been described as an invasive parasite with case reports from sika deer in both Austria and Germany, but it is also known that it has been established among wild roe deer, fallow deer and red deer in the Czech Republic [9].

Another difference compared to the study by Beaumelle et al. [15] is that we did not identify *B. trigonocephalum*. However, in France this species was only found at a low relative abundance in one locality. Furthermore, unlike Beaumelle et al. [15], we detected *Mazamastrongylus dagestanica*, which, like *S. houdemeri*, has its origins in the Caucasus region. *Mazamastrongylus dagestanica* was formerly known as *Spiculopteragia alcis* and was then considered a typical parasite in roe deer and moose, although the original morphological description was performed on specimens from sheep [63]. In our study, this species was found exclusively in roe deer (9%). Interestingly, in an older Swedish study from the 1970s, the prevalence of *S. alcis* was 38% in roe deer and 100% in moose [24]. In addition, we identified nine strongyle species usually found in horses. However, when the data are combined, these taxa represent only 1.4% of the total number of reads. Thus, this may be due to either laboratory contamination or sequencing artifacts.

Although there are few alternative cost-effective ways of sampling and objective identification of wildlife nematodes, as stated by Beaumelle et al. [15], a disadvantage with the nemabiome approach is that sequence data for wildlife nematodes are either missing or highly underrepresented in common databases. This is also evident in our analysis, where the different species are represented by between 1 and 173 ASVs (Table 1). The two species with the most ASVs in our study are *O. leptospicularis* and *T. axei,* while *O. ostertagi* and *Cooperia* sp. by only two and one ASV(s), respectively. Furthermore, like Beaumelle et al. [15], we were unable to identify the species for some ASVs. Still, in our study, as many as 26 of 31 (84%) ASV clusters were assigned to the species level. Nonetheless, the taxonomy of one of the more common species we found in roe deer, *Trichostrongylus* sp. B is not entirely clear and it therefore needs further investigation. Regardless, nemabiome sequencing is a valuable method for the objective assessment of the diversity and richness of wildlife nematodes, even if the method is not free from drawbacks. For example, in some cases proper identification to species failed because reference sequences were missing in the public databases (i.e. one member each in genera *Dictyocaulus*, *Cooperia* and *Ostertagia* and two within genus *Trichostrongylus*).

Another limitation in our study is that the number of samples per host examined varied a lot in both mouflon (*n* = 13) and red deer (*n* = 18), being less well studied than fallow deer (*n* = 106) and roe deer (*n* = 125). Thus, due to the low number of samples from some host species, we cannot rule out that we missed some species, such as *H. contortus* in mouflon. It is also well known that the susceptibility to nematode infections differs both between and even within the same host species. For example, in France adult males had heavier infections compared to juveniles and adult females [64]. Similarly, in red deer in central Spain both the occurrence and intensity of abomasal parasitism were higher in older animals, particularly in males [65]. However, only sex showed an impact on the nematode burden in roe deer during the hunting season on the northwest of the Iberian Peninsula with higher burdens in males [20]. In contrast, fallow deer calves had significantly higher worm counts than yearlings but there was no difference between sexes [60]. Similarly, the alpha diversity of parasite communities in roe deer did not differ between sexes in France [15]. In addition, there are seasonal trends in fecal egg counts. For example, a bimodal pattern for intensity of infection by gastrointestinal nematodes was observed in fallow deer on the Iberian Peninsula [65]. Thus, absence or low occurrence of some species could equally be because of hypobiosis. On the other hand, neither EPG nor the amount of sample for coprocultures affected the species abundance. Nevertheless, since the samples we investigated were mainly obtained from hunters during the hunting season in autumn it cannot be ruled out that the season affected the outcome. Nonetheless, we identified 31 species, where the majority (68%) occurred in more than one type of host.

## Conclusions

In this study we studied the nemabiomes of four wildlife hosts in Sweden. We found that *T. axei* was the most commonly identified species, contrasting with our previous study on sheep, where *H. contortus* and *T. circumcincta* were the most abundant. Based on our findings, we can conclude that wild animals in Sweden are infected with species that theoretically can be transmitted to sheep. However, we assess that the risk of this happening is low as the nemabiome profiles between host species are so different. In addition to several typical wildlife nematodes, the invasive parasite *S. houdemeri* was found for the first time in Sweden in fallow deer, red deer and mouflon while *Ashworthius sidemi* was absent. We conclude that nemabiome analysis is a powerful tool since we were able to identify 31 species. A few could not be assigned to species level. For the future, it is therefore important/needed to sequence morphologically identified specimens to further improve species delineation using nemabiome analysis approach in wildlife.

## Data Availability

The datasets used and/or analyzed during the current study are available from the corresponding author on reasonable request. The raw ITS2 data are available in the BioStudies database (http://www.ebi.ac.uk/biostudies) under accession number S-BSST527.
